# Supplementary material for: The effects of temporal cues, point-light displays, and faces on speech identification and listening effort
Source: PLoS One. 2023 Nov 29;18(11):e0290826. doi: 10.1371/journal.pone.0290826 (PMC10686424; doi:10.1371/journal.pone.0290826)
Supplement: S1 File — (PDF) [file pone.0290826.s001.pdf]

Supporting Information for *The Effects of Temporal Cues, Point-Light Displays, and Faces on Speech Identification and Listening Effort*

Main Experiment

Word identification accuracy

Table S1. Mean and standard deviations for word identification by visual condition and noise type.

	**Energetic**	**Informational**
**audio-only**	42.38 (35.62)	45.73 (38.48)
**temporal**	43.84 (35.64)	44.40 (38.47)
**point-light**	46.70 (36.49)	47.24 (39.23)
**natural face**	65.37 (35.15)	71.10 (34.44)

Listening Effort

Table S2. Summary data for listening effort ratings by visual condition and noise type.

	**Energetic**	**Informational**
**audio-only**	86.38 (16.75)	86.74 (14.99)
**temporal**	85.90 (16.89)	85.23 (17.71)
**point-light**	85.41 (16.44)	84.57 (17.54)
**natural face**	78.72 (20.87)	76.47 (20.72)

Pilot Study

Prior to running the main experiment reported in the associated manuscript, *The Effects of Temporal Cues, Point-Light Displays, and Faces on Speech Identification and Listening Effort*, we ran a version of the study that was nearly identical but had easier SNRs: -5 for the information masker and -7 for the energetic masker. The results of this pilot study were qualitatively similar to those reported in the main experiment, but the data suffered from a ceiling effect in the informational masker that complicated interpretation of the results. For example, the point-light display improved intelligibility relative to audio-only speech in the energetic but not the informational masker, but because difficulty was confounded with masker, it is not clear whether this finding occurred because point-light displays provide information that selectively overcomes energetic—but not informational—masking or whether point-light displays only help at difficult SNRs.

Thus, we opted to rerun the study with more difficult SNRs and that is what is reported in the main paper. We report the results of the pilot study here for completeness. The procedures and conventions for data analysis were otherwise identical to the main experiment.

Energetic Masking

A model that included a fixed effect for visual condition provided a better fit for the data than one that did not, $\chi^2_3 = 192.6$, $p < .001$. Word identification accuracy was poorer in the audio-only condition than the point-light ($B = 0.17$, $SE = 0.06$, $z = 2.91$, $p = .004$) and natural face ($B = 1.31$, $SE = 0.09$, $z = 15.28$, $p < .001$) conditions, but the audio-only condition and the temporal condition did not differ ($B = 0.04$, $SE = 0.05$, $z = 0.82$, $p = .41$). Identification accuracy for the point-light condition was better than the temporal-only condition ($B = -0.13$, $SE = 0.05$, $z = -2.55$, $p = .01$) and poorer than the natural face ($B = 1.14$, $SE = .08$, $z = 15.07$, $p < .001$). Thus, the point-light display (but not the temporal cue) provided modest audiovisual benefit, but enhancement was greatest for the natural talking face.

Informational Masking

A model that included a fixed effect for visual condition provided a better fit for the data than one that did not $\chi^2_3 = 150.65$, $p < .001$. Word identification accuracy was poorer in the audio-only condition than natural face condition ($B = 1.38$, $SE = 0.11$, $z = 13.05$, $p < .001$), but rates of identification accuracy did not differ for the audio-only and temporal conditions ($B = .04$, $SE = .07$, $z = .58$, $p = .56$) or audio-only and point-light conditions ($B = .05$, $SE = 0.10$, $z = .50$, $p = .61$). Identification accuracy in the point-light condition did not differ from the temporal condition ($B = -.01$, $SE = .09$, $z = -.08$, $p = .94$), but was poorer than the natural face ($B = 1.33$, $SE = .11$, $z = 12.56$, $p < .001$). Thus, unlike in the energetic masking condition, the only stimulus to provide audiovisual enhancement was the natural face.

Table S3. Mean and standard deviations for word identification accuracy (in percent correct) by visual condition and noise type.

	**Energetic**	**Informational**
**A-only**	58.32 (35.90)	70.43 (36.02)
**Temporal**	59.45 (35.45)	71.69 (34.75)
**Point light**	61.91 (35.23)	70.94 (35.60)
**Natural Face**	78.27 (30.35)	87.94 (23.42)

Comparing Maskers. Next, we subsetted the data to include only the audio-only and natural face conditions and combined the data from the energetic and informational maskers to assess whether masker type moderates the magnitude of audiovisual benefit. The full model contained fixed effects for visual stimulus, masker type, and the interaction. The reduced model was identical to the full model but omitted the interaction to assess whether audiovisual benefit differed across maskers. The full model provided a better fit for the data than the reduced model ($\chi^2_1 = 15.64$, $p < .001$), and the summary output for the full model also indicated a significant interaction ($B = -.23$, $SE = 0.06$, $z = -4.00$, $p < .001$). In contrast to previous research (Helfer & Freyman, 2005), participants showed moderately more audiovisual benefit with energetic maskers (a 19.95% change) than informational ones (a 17.51% change).

Listening Effort

The listening effort analyses mirror those of the word identification analyses, but we did not model item-level random effects because the NASA assessments are completed after blocks of sentences rather than after individual items.

Energetic masking. A model that included a fixed effect for visual condition provided a better fit for the data than one that did not ($\chi^2_3 = 44.67$, $p < .001$). Participants reported less subjective effort in the natural face condition than the audio-only condition ($B = -9.86$, $SE = 1.55$, $t = -6.35$, $p < .001$) and the point-light condition ($B = -8.74$, $SE = 1.32$, $t = -6.61$, $p < .001$), but all other preregistered comparisons were nonsignificant: audio-only vs. temporal ($B = .35$, $SE = .99$, $t = .35$, $p = .72$), audio-only vs. point-light ($B = -1.12$, $SE = 1.18$, $t = -.95$, $p = .35$), and temporal vs. point-light ($B = 1.47$, $SE = .88$, $t = 1.66$, $p = .10$).

Informational masking. A model that included a fixed effect for visual condition provided a better fit for the data than one that did not, $\chi^2_3 = 69.33$, $p < .001$. Participants reported less subjective effort in the natural face condition than the audio-only condition ($B = -12.53$, $SE = 1.54$, $t = -8.11$, $p < .001$) and the point-light condition ($B = -11.16$, $SE = 1.40$, $t = -7.95$, $p < .001$), but all other preregistered comparisons were nonsignificant: audio-only vs. temporal ($B = -.02$, $SE = 1.11$, $t = -.02$, $p = .98$), audio-only vs. point-light ($B = -1.37$, $SE = 1.12$, $t = -1.23$, $p = .22$), temporal vs. point-light ($B = 1.35$, $SE = .98$, $t = 1.37$, $p = .17$).

Table S4. Summary data for listening effort ratings by visual condition and noise type.

	Energetic	Informational
A-only	80.17 (21.58)	74.66 (22.52)
Temporal	80.52 (20.58)	74.64 (21.82)
Point light	79.05 (20.81)	73.29 (21.51)
Natural Face	70.31 (24.63)	62.13 (26.58)

Comparing maskers. Finally, we assessed whether the reduction in subjective effort from adding a talking face was greater for informational than energetic masking. The full model contained fixed effects for visual condition (audio-only vs face), masker type (energetic vs informational), and the visual condition by masker type interaction, with random by-participant slopes for masker and condition. The reduced model that did not include the interaction term and resulted in a poorer fit for the data than the model that did contain the interaction $\chi^2_1 = 4.78$, $p = .03$. In the energetic masking condition, the addition of a natural face decreased self-reported effort by 9.86 points (on a scale from 0–100) whereas the change for informational was 12.53 points. Previous work has shown greater audiovisual benefit for informational masking than energetic masking in terms of identification accuracy, but this is the first to show the effect also holds for self-reported listening effort.
