# Supplementary material for: The effects of temporal cues, point-light displays, and faces on speech identification and listening effort
Source: PLoS One. 2023 Nov 29;18(11):e0290826. doi: 10.1371/journal.pone.0290826 (PMC10686424; doi:10.1371/journal.pone.0290826)
Supplement: Attachment — Submitted filename: Response to reviewers.pdf [file pone.0290826.s002.pdf]

We thank the editor and the reviewers for the time and energy that went into reviewing this paper! We've addressed the issues raised in the reviews and summarize the changes below (in bold).

Journal Requirements:

When submitting your revision, we need you to address these additional requirements.
We have addressed all the journal requirements. The individual in figure 1 is the second author of the paper. The consent form seems intended for use by participants rather than by authors (e.g., it says " have discussed this consent form with ___, who is an author of this article"). Therefore, it doesn't seem appropriate to have her sign the consent form or reference it in the text.

Reviewers' comments:

Reviewer #1: Summary

This study tested the degree to which different types of visual information (full face, point-light lips, and a dot that modulates size) impact audiovisual speech identification and listening effort when the auditory signal is partially masked by energetic or informational noise. The results suggest that a full face provides the most visual benefit to audiovisual speech identification, with a point-light display of the talker's lips providing a benefit in energic masking but not informational masking. In contrast, the modulating dot provided no visual benefit in either masking condition. Compared to the auditory only condition, visibility of the full-face reduced listening effort across masking conditions. Point light lips and the dot display did not affect listening effort in the energetic masking condition, but both reduced effort in the informational masking condition, somewhat in contrast with identification performance.

General Comments

The manuscript is well written, presenting clear motivation and hypotheses for the roles of different types of visual information and auditory masking that can affect audiovisual benefit and listening effort. The methods are largely sound, the results are clearly presented, and the interpretation of the results is mostly reasonable. I have only a couple points of concern that the authors should consider pertaining to their description of their conditions and the interpretation of their results that should be addressed before publication.

The authors describe their dot display as preserving temporal information. However, it can be argued that this display does not preserve all temporal information. Modulation of the dot's size is determined by the time-varying oral aperture from the full face display, but aperture alone does not necessarily preserve all salient time-varying information available from a speakers articulating face. Additionally, and as the authors state in the introduction, oral aperture can

relate to vocal effort and amplitude, meaning that the time-varying size of the dot can carry more than just temporal information. The authors should be clear about what information may be available from their dot display, and how information varies across their visual conditions (e.g., full face: all available visible information; point-light: isolated kinematic information for lip movements; dot display: non-kinematic information for oral aperture and vowel intensity information).
Great points, thank you! We've added text regarding this in the "current study" section.

Additionally, the point-light display used in this study isolated points to the speaker's lips, which can provide kinematic information for lip movements (intrinsically linked to lip aperture). However, previous studies have found that the number and placement of dots (i.e., lips, teeth, tongue, and surrounding areas) can affect the amount of kinematic information available from a point-light display and its effect on audiovisual speech perception (e.g., Rosenblum et al., 1996). I'm sure the software used to create the point-light displays for this study limits the placement and saliency of points derived from an articulating face, but the authors should consider how varying the number of points may affect their results.
Another good point. We've added a comment about that in the discussion

Reviewer #2:

I have no major issues with the paper. The work seems carefully done, the data properly analyzed, and the paper clearly written. I have just a few comments that I hope will improve the manuscript before publication.
Thank you so much for these kind words!

First, a quick request for adding line numbers in the future (for journals that do not auto-add them on submission, since two sets of line numbers is worse than none).
This is a great point, thanks for the suggestion!

In the intro I am missing a few references to to two of Yi Yuan's studies:
https://pubs.aip.org/asa/jasa/article/147/3/EL246/997260/Visual-analog-of-the-acoustic-amplitude-envelope
https://pubs.asha.org/doi/abs/10.1044/2021_JSLHR-20-00688
We've incorporated these citations. Thanks!

It also might make sense to include Fiscella et al 2022 (https://link.springer.com/article/10.3758/s13414-022-02440-3), which compares AV benefit of a visual face to an upside down visual face (such that timing is preserved but linguistic information is partially disrupted).
Given the relatively large body of work assessing the extent to which variations in the signal quality of the talking face affect audiovisual benefit (e.g., faces with varying degrees of visual blur, upside down faces, small faces, etc.), and our desire to keep this paper short, we have opted not to include this or other similar citations in this paper.

Page 8, informational maskers: was each babble stream presented at -8, or was the sum of the streams at -8 dB SNR? In the former case, the total SNR would be about -11 dB.
Good catch! The sum of the streams was presented at -8 SNR. We've made this change.

Page 8, second line of Procedures: binaurally --> diotically, unless there were binaural spatial cues
Done!

Page 9-10: the first two paragraphs of your results feel to me like they belong in the methods section.
We prefer to keep the information about analyses and data cleaning in the Results section, near where the outcome of those analyses are described.

Page 15, first paragraph: It may also just have some additional 2D noise. Did the authors visually inspect the dots overlaid on talker videos both in video and individual frames? There are *many* models for generating these landmarks, some better than others, and I am not familiar with the one used here. The dlib default, for instance, is quite bad in my experience. This one (https://twitter.com/alexcarliera/status/1678720831122186240) seems to be really excellent (I think that may be the first time I've referenced a tweet in a review). I am not sure if it is available for use or not, but it's an example of what's possible.
HA! We appreciate the Tweet (sorry, the X?). We did look at the videos and individual frames, but without another method for generating landmarks to compare it to, it's difficult to know how accurately the point light displays are representing the facial movements. We added a sentence about this in the Discussion section and suggest that comparing multiple methods for generating landmarks might be informative, for exactly the reason you suggest.

Page 16, second paragraph: The connection between the results of Helfer and Freyman and this study would depend on the slope of the psychometric function, correct? (which is not the subject of either study). In their fig. 1 they show percent correct for three SNRs, one of which is -8 dB (used also in your study). To my eye, the gap between F-F A and F-F AV PC seem to differ by about 4% between the two plots.

While I agree your effect is small, I am a little uncomfortable with saying it is much smaller than the Helfer and Freyman effect, which also seems small from their figures and is reported in different units (unless I'm misunderstanding their results, which is entirely possible!).
It's certainly true that the effect was relatively small looking at an SNR of -8, but the AV benefit is much larger at an SNR of -4, which rendered intelligibility levels comparable to those observed in this study. However, we've toned down the language to reflect the fact that our findings weren't *much* smaller than those observed by Helfer and Freyman (2005).
